# Emergency department presentations for atrial fibrillation and flutter in Alberta: a large population-based study

**DOI:** 10.1186/s12873-016-0113-2

**Published:** 2017-01-10

**Authors:** Rhonda J. Rosychuk, Michelle M. Graham, Brian R. Holroyd, Brian H. Rowe

**Affiliations:** 1 Department of Pediatrics, University of Alberta, Rm 3-524, Edmonton Clinic Health Academy (ECHA) 11405 87 Avenue NW, Edmonton, AB T6G 1C9 Canada; 2 Women & Children’s Health Research Institute, Edmonton, Canada; 3 Department of Medicine, University of Alberta, University of Alberta Hospital, 2C2 Walter Mackenzie Building, 8440 112 Street, Edmonton, AB T6G 2B7 Canada; 4 Department of Emergency Medicine, University of Alberta, University of Alberta Hospital, 1G1.42 Walter Mackenzie Building, 8440 112 Street, Edmonton, AB T6G 2B7 Canada; 5 Alberta Health Services, Edmonton, Canada; 6 School of Public Health, University of Alberta, Edmonton, Canada

**Keywords:** Administrative databases, Atrial fibrillation, Atrial flutter, Emergency department

## Abstract

**Background:**

Atrial fibrillation or flutter (AFF) are not infrequent presenting problems in Emergency Departments (ED); however, little is known of the pattern of these presentations. This study provides a description of AFF presentations and outcomes after ED discharge in Alberta.

**Methods:**

Provincial administrative databases were used to obtain all primary ED encounters for AFF during 1999 to 2011 for patients aged >35 years. Data extracted included demographics, ED visit timing, and subsequent visits to non-ED settings. Analysis included summaries and standardized rates.

**Results:**

During the study period, there were 63,398 ED AFF visits from 32,104 distinct adults. Median ages for females and males were 75 and 67 years, respectively; more men (52%) and patients > 65 presented. Overall, the standardized rates remained similar (2.8 per 1,000 over the study period). Specific populations of human services recipients and First Nations had higher ED visit rates for AFF than other groups. Predictable daily, weekly, and monthly trends were observed. The ED visits were followed by numerous subsequent visits in non-ED settings; however, First Nations and women had lower rates of specialist follow-up.

**Conclusions:**

Annually, over 5,000 ED presentations of patients experiencing AFF occur in Alberta and admissions proportions are declining. While presentation rates across the province are stable, follow-up with physicians, consultation with cardiologists and health outcomes vary based on socio-economic, age, sex, and First Nations status. Further research is required to understand the causes and consequences of these inequalities and to standardize care.

**Electronic supplementary material:**

The online version of this article (doi:10.1186/s12873-016-0113-2) contains supplementary material, which is available to authorized users.

## Background

Atrial fibrillation and flutter (AFF) are the most common clinically diagnosed arrhythmias affecting older populations (approximately 350,000 Canadians [1] and 2.66 million Americans) [2]. These arrhythmias are associated with an increased long-term risk of stroke, heart failure, and all-cause mortality [3]. The age-sex-adjusted prevalence of AFF has increased over the years (from 613 per 100,000 population in 2000 to 1,148 per 100,000 population in 2005 in Alberta, Canada [4]), attributed to an aging population [5] and a rising prevalence of chronic heart disease [6, 7].

In Emergency Departments (EDs), physicians often manage patients with either recent-onset (first detected or paroxysmal) or permanent (chronic) AFF. Most patients with acute AFF are managed by physicians staffing emergency departments in Alberta. The large majority are seen by the full-time emergency physicians (in urban and regional EDs) or on-duty physicians staffing smaller hospital emergency departments (in rural EDs). Moreover, consultations are required for all admissions to urban and regional hospitals. The epidemiology of AFF in the ED setting is not well known and, what is known, arises from United States (US) data [8] and one recent Ontario study [9]. This paper describes patients who presented to EDs for AFF using Alberta’s population-based databases. In particular, we focus on characteristics (e.g., age, sex), key process of care features of the ED presentation (e.g., triage level), and outcomes including physician follow up and mortality.

## Methods

### Setting

This cohort study used data from the Ambulatory Care Classification System (ACCS) database in the province of Alberta, Canada during April 1, 1999, to March 31, 2011. Since the Canadian health care system is a public, single payer system, the ACCS database captures 100% of the presentations by Albertans made to the ED.

The ACCS database [10] tracks ambulatory care visits, such as ED presentations, within government-funded health facilities in Alberta and supplies data for Canada’s National Ambulatory Care Reporting System [11]. Patient encounters in all 104 provincial EDs are entered into computerized abstracts that constitute the majority of records. Using a uniform protocol, trained medical records nosologists code each ED chart using International Classification of Diseases (ICD): ICD-9-CM [12] (prior to April 1, 2002) or ICD-10-CA [13] (April 1, 2002 onward) codes. Each ACCS record represents a unique service and contains visit start and end dates and times, diagnoses, and disposition status. Demographic data were obtained by linking individuals in ACCS to the annual Alberta Health Care Insurance Plan cumulative registry file and follow-up physician visits to non-ED settings were obtained from the Physician Claim File. Alberta Vital Statistics provided data on deaths.

### Protocol

Diagnostic information in ACCS consisted of a main ambulatory diagnosis field, and up to 9 additional fields. To be considered an ED presentation for acute AFF, the *main* (*first*) diagnosis field had to have diagnostic codes 427.3x “Atrial Fibrillation and Flutter” (includes 427.31 and 427.32) or I48.x “Atrial Fibrillation and Flutter” (includes I48.0 and I48.1). ED presentations during the study were extracted for Alberta residents who matched the case definition and whose age at the ED presentation was ≥35 years.

Demographic variables extracted were age in years at ED presentation (grouped as 35–39,…,80–89, 90+; seniors defined as age ≥65), sex (male or female), and geographic area of residence (i.e., North, Edmonton, Central, Calgary, South). Alberta is administratively organized into five geographic zones (i.e., North in the North of the province, Central in the central area of the province, and South in the southern tip of the province, along with two major urban centres (Edmonton and Calgary area) for the delivery of health care services [14]. The administrative data sources do not include a measure of socio-economic status; however, a proxy measure can be created based on some payment data. The Alberta government funds health care in the province and healthcare insurance premiums provided partial funding through January 1, 2009. Residents with lower incomes or on social services were eligible for premium subsidies the government classified as individuals as receiving Government Sponsored Programs or as a Human Services Recipient. These classifications only apply to individuals aged less than 65 and thus, would not apply to seniors. Also, many Aboriginal individuals have “Treaty” status based on treaties with the Canadian government [15] and these individuals are identifiable in the database because the Canadian government, rather than the provincial Alberta government, provide health care funding. Using these classifications and the age of the individual, a proxy measure for socio-economic status can be obtained to create six mutually exclusive groups based on age and subsidy. For those aged 35–64 years, the groups: First Nations (i.e., Aboriginal Canadians with Treaty status), Government Sponsored Programs, Human Services Recipient, and Other were created. For seniors (i.e., age ≥ 65 years), patients are categorized as First Nations or Non-First Nations. All of these variables were the demographic variables available in the Alberta Health Care Insurance Plan cumulative registry file.

The ED data included the start and end dates and times for the presentation. Triage level represents the urgency of the presentation and is based on the Canadian Triage and Acuity Scale (CTAS) [16]. The five categories are: resuscitation (level I, see patient immediately), emergency (level II, see patient within 15 min), urgent (level III, see patient within 30 min), semi-urgent (level IV, see patient within 60 min), and non-urgent (level V, see patient within 120 min). Mandatory triage level reporting was implemented at different times in different EDs within Alberta. All patients departing an ED are given a disposition classification. Patients admitted were defined as those admitted to the facility (including those to the Critical Care Unit or Operating Room) or transferred to another acute care facility [10]. Patients were also classified as discharged or other (e.g., death, left without being seen).

Physician visits to non-ED settings (follow-up visits hereafter) for any diagnosis were also extracted if they occurred within 365 days of the ED presentation. Data included the date, specialty of physician (grouped as specialist [cardiology or internal medicine] or other), and facility type (active treatment hospital, practitioner’s office, or other). Physician visits in the 365 days prior to the ED visit were similarly extracted and used to determine comorbidities according to a standard coding scheme [17].

### Data analysis

Summaries included frequencies, percentages, medians and interquartile ranges (IQRs). Yearly age group-specific rates per 1,000 population (≥35 years) and sex-age group directly standardized visit rates (DSVRs [18]) were calculated. The 1999/2000 Alberta (≥35 years) population stratified by age group and sex was the reference population and 95% confidence intervals (CIs) were obtained. The Cochran-Armitage trend test was used to assess time trends in admission rates. When results were similar across years, more detailed summaries for the last year of data were provided. Monthly and day of week patterns were not statistically analyzed as these results were intended to be descriptive rather than inferential. Data were analyzed using TIBCO Spotfire S+ (Version 8.1.1 for Linux, TIBCO Software Inc, Palo Alto, CA. 2008) and SAS (Version 9.2, SAS Institute Inc. Cary, NC. 2010).

For analyses with both ED and follow-up visits, a discharged subset of “index” ED visits was created with one record per individual discharged from the ED. One ED visit was randomly selected among the multiple presentations from the same individual (using computer implemented simple random sampling), to enable index visits from across the study period. The times from the index ED visit to the first follow-up visit were calculated and displayed with Kaplan-Meier curves. Time was censored at 365 days after discharge or the next ED visit. Estimated median times are reported with 95% CIs and log-rank tests compare groups. Follow-up visits were summarized within 30 and 90 days of ED discharge. Deaths within 30 and 90 days of ED discharge were summarized. These summaries where intended to be descriptive rather than inferential. Deaths were classified as AFF-related if the cause of death matched the AFF case definition.

## Results

### General trends

**Fig. 1:**
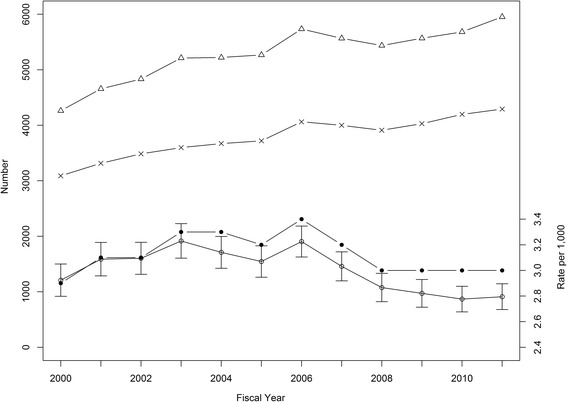
ED visits for AFF made by patients (age ≥35 years) and rates per 1,000 population in Alberta by fiscal year: left axis: number of ED visits (∆), patients with ED visits (+); right axis: crude rates (●), sex and age group directly standardized visit rates (○) with 95% confidence intervals

During the 12 year study period, 63,398 (0.6%) of all ED visits were for AFF and ED visits increased from 3,090 in 1999/2000 to 5,953 in 2010/2011 (Fig. 1). The 63,398 visits were made by 32,104 individual patients. On average, there were 2 visits/patient (median = 1, IQR = 1 to 2, max = 53). Most individuals (20,396; 63.5%) presented only once to an ED and the rest had multiple visits (e.g., 5,699 [17.8%] and 2,361 [7.4%] presented two and three times, respectively). The crude ED presentation rate remained stable at 2.9 to 3.4 per 1,000.

### Age and sex

**Fig. 2:**
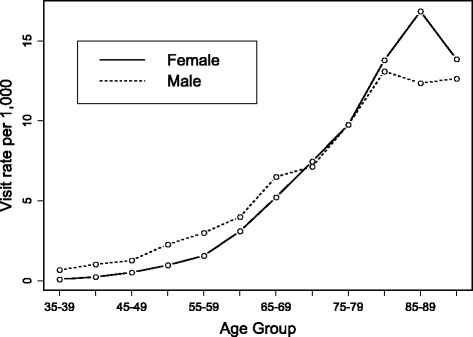
Age-specific ED visits for AFF per 1,000 population in 2010/2011 for males and females

Among 63,398 ED presentations, 48.2% were made by females. Younger patients were more likely to be male (e.g., the rate ratio for female vs male for age 65–69 was 0.80, 95% CI 0.69 to 0.93, *p* = 0.003) and older patients were more likely to be female (e.g., the rate ratio for age 85–89 was 1.36, 95% CI 1.13 to 1.66, *p* = 0.001; Fig. 2). The DSVRs were similar for each year and close to the crude rates (Fig. 1).

### Special populations

In 2010/2011, the majority of ED visits for AFF were made by non-First Nations seniors (3,697, 62.1%; Table 1). First Nations seniors also had approximately three times as many ED presentations as would have been expected by the population proportion. Conversely, the Other aged 35–64 years group represented 70.3% of the population but had disproportionately fewer ED presentations (29.8%). The DVSRs for seniors were highest for First Nations (11.5 vs 8.9 per 1,000; Additional file 1 Table S1) and for those aged 35 to 64 years, the First Nations group had the highest DSVRs (2.3 vs 1.7, 1.5, and 1.1). These patterns were seen for other study years and DSVRs were relatively stable across the study period (Additional file 1: Figures S1 and S2).

**Table 1:** Population (aged ≥35), number of ED visits, and number of unique individuals for each subsidy group in 2010/2011

	Alberta Population (%)		No. ED Visits for AFF (%)		No. Individuals with ED Visits for AFF (%)	
First Nations aged 35−64	42,409	(2.2)	92	(1.5)	68	(1.6)
Government Sponsored Programs aged 35−64	62,040	(3.2)	206	(3.5)	128	(3.0)
Human Services Recipient aged 35−64	65,230	(3.3)	133	(2.2)	95	(2.2)
Other aged 35−64	1,373,453	(70.3)	1,773	(29.8)	1,253	(29.2)
First Nations Seniors	4,914	(0.3)	52	(0.9)	38	(0.9)
Non-First Nations Seniors	405,932	(20.8)	3,697	(62.1)	2,716	(63.3)

### Triage level

By 2010/2011, the majority (79.9%) of ED visits for AFF were classified as either CTAS II emergency (42.3%, 2,516) or CTAS III urgent (37.7%, 2,243) (Additional file 1: Table S2).

### Visit timing

**Fig. 3:**
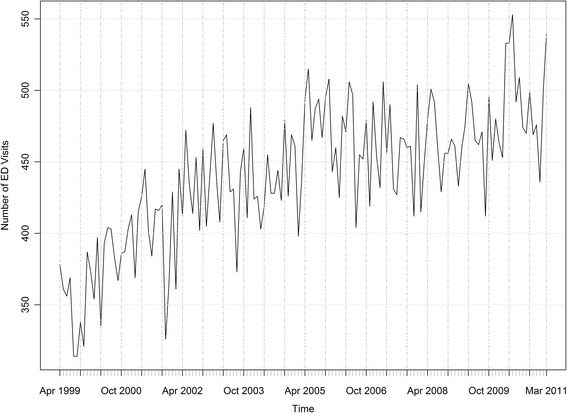
ED visits for AFF by month

ED visits for AFF ranged from 314 per month (August, September of 1999) to 553 per month (May 2010), with a median of 445. Similar monthly patters were seen for each fiscal year (Fig. 3).

The number of ED visits varied with the day of week. Monday (9,895, 15.6%) and Tuesday (9,708, 15.3%) tended to have more visits than the other days while Saturday (7,875, 12.4%) and Sunday (7,699, 12.1%) had less visits. The patterns of ED visits by hour of the day were similar for each fiscal year. Generally ED visits peaked between 7 am and 12 pm and were less frequent between 12 am and 6 am.

Of the 59,205 visits (93.4%) with start and end date and time information, the median total ED length of stay (LOS) was 3h50m (Additional file 1: Table S3). For admissions (17,776 visits) and ED discharges (41,219 visits), the median ED LOS was 4h16m and 3h43m, respectively. The remaining 210 visits had other dispositions. The Edmonton and Calgary Zones, corresponding to the urban areas where ED volumes are highest, had longer LOSs than the other Zones.

### Disposition

**Fig. 4:**
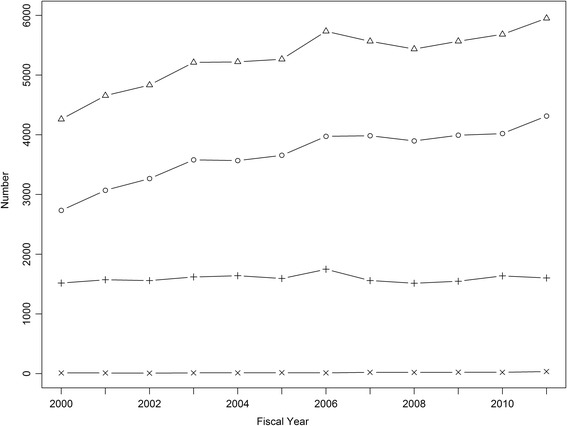
ED visits for AFF by disposition and fiscal year: visits (∆), discharged (○), admitted (+) and no completion of care (×)

Discharge was the most common disposition and there was an increasing trend of ED discharge over the study period (Fig. 4, Cochran-Armitage trend test, *p* < 0.001). For 2010/2011, 4,314 (72.5%) ED visits for AFF ended in discharge and 1,602 (26.9%) in admissions.

### Discharged subset

There were 44,064 ED visits made by 21,062 individual patients that concluded with a discharge. For each of the 21,062 patients, we used simple random sampling to select a single visit, comprising the discharged subset group. The discharged subset had 10,007 (47.5%) females and a mean age of 69.0 years. Overall, 65% (13587/21062) and 28% (5,864) were from the non-First Nations seniors and Other 35–64 group, respectively. Using physician visits in the 365 days prior to the ED visit, the most common comorbidities documented were hypertension (9,266 patients, 44.0%), cardiac arrhythmias (8,058, 38.3%), congestive heart failure (3,152, 15.0%), chronic pulmonary disease (2,825, 13.4%), and depression (2,140, 10.2%).

#### Prior physician claims for AFF

At least one physician claim for AFF in the year prior to the ED visit was documented for 7,111 (33.8%) of the 21,062 patients. There were 3,136 and 5,111 physician claims for AFF in the 7 and 30 days prior to the ED visit, respectively. Most were made by the non-First Nations seniors group (1,931 [61.6%] at 7 days; 3,197 [62.6%] at 30 days).

#### Return ED visits

**Fig. 5:**
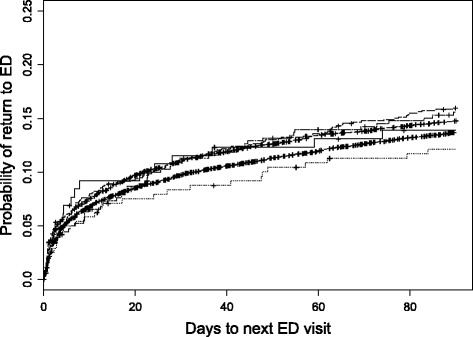
Time to return ED visit by subsidy group. At 90 days, from top to bottom are: Government Sponsored Program 35–64, Human Services Recipient 35–64, Other 35–64, First Nations Seniors, Non-First Nations Seniors, and First Nations 35–64

At 30 days after ED discharge, 2,138 (10.3%, *n* = 20,854) patients had at least one return ED visit for AFF. At 90 days, the number increased to 2,949 (14.3%, *n* = 20,599). The time to ED return varied among subsidy groups (Fig. 5; *p* < 0.001), with the non-First Nations seniors and First Nations aged 35–64 groups having the longest times.

#### Follow-up visits

**Fig. 6:**
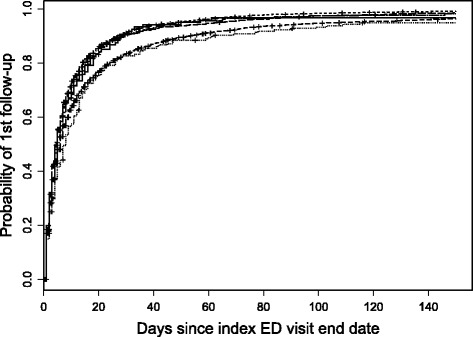
Time to first follow-up visit by subsidy group after discharge from the ED. Top 4 lines represent Human Services Recipient 35–64, Government Sponsored Programs 35–64, First Nations Seniors, and Non-First Nations Seniors. Bottom 2 lines represent Other 35–64 and First Nations 35–64

At 30 and 90 days after ED discharge, 85 and 92% of patients had at least one follow-up visit. The estimated median time to the first follow-up visit was 5 days (95% CI: 5 to 5). The estimated median times differed by subsidy group (*p* < 0.001, Fig. 6) although the variation is likely not clinically important: the First Nations 35–64 group was the longest at 8 days (95% CI: 6 to 10) whereas the other groups were 4 days (Government Sponsored Programs 35–64, 95% CI: 4 to 5), 5 days (First Nations seniors, 95% CI: 4 to 7; non-First Nations seniors, 95% CI: 4 to 5; Human Services Recipient 35–64 groups, 95% CI: 4 to 6), and 6 days (Other 35–64, 95% CI: 6 to 6).

#### Specialist follow-up visits

**Fig. 7:**
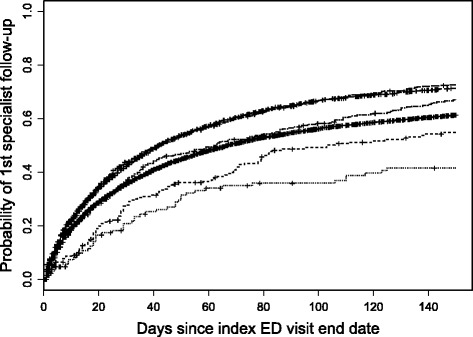
Time to first follow-up visit with a specialist by subsidy group after discharge from the ED. At 150 days, from top to bottom: Government Sponsored Programs 35–64, Other 35–64, Human Services Recipient 35–64, Non-First Nations Seniors, First Nations 35–64, and First Nations Seniors

At 30 and 90 days after ED discharge, 38 and 58% of patients had a specialist follow-up visit, respectively. The estimated median time to a specialist follow-up visit was 56 days (95% CI: 54 to 59) and these times differed by sex (*p* < 0.001) and subsidy group (*p* < 0.001). Females had slightly longer estimated median times (61 days, 95% CI: 57 to 64) than males (53 days, 95% CI: 50 to 55). The Human Services Recipient and Other 35–64 groups had the shortest times of 42 days, 95% CIs: 35 to 50 and 40 to 44, respectively (Fig. 7). The First Nations groups had the longest times: 103 and 182 days for those under and over age 65, respectively. Generally, patients with more urgent care requirements (triage level) had shorter times to see a specialist than those requiring less urgent care (*p* < 0.001). For example, the estimated median time was 40 days (95% CI: 38 to 42) for patients triaged as emergency whereas it was 102 days (95% CI: 91 to 121) for semi-urgent patients.

#### Deaths after ED discharge

At 30 days after the ED visit, 234 (1.1%) of patients had died and 14 (6.0%) of the deaths were classified as AFF-related. At 90 days, there were 548 (2.6%) deaths and 24 (4.4%) were AFF-related. There were more deaths for females and the vast majority of deaths occurred in seniors that were non-First Nations (*n* = 13,587): 203 (1.5%) and 476 (3.5%) deaths at 30 and 90 days, respectively.

## Discussion

Using a population-based database and extensive linkages among databases, this study provides a comprehensive evaluation of AFF in one Canadian province. This study has several key findings. First, AFF is a not infrequent ED presentation but less common than other cardio-respiratory conditions (i.e., COPD [19] asthma [20]); however, this analysis under-estimates the magnitude of AFF in the ED. Since inclusion was restricted to patients with the first diagnosis being AFF, many chronic cases of AFF would not be included (e.g., if patients primarily presented with other acute cardiac [acute coronary syndrome, heart failure] or respiratory [chronic obstructive pulmonary disease] conditions as their primary concern, AFF would be coded as a secondary cause of presentation). Second, the seasonal trends and the day and hour trends in AFF are similar to other chronic conditions in Alberta such as asthma [20] and COPD [19]. Third, times to follow-up varied considerably across socioeconomic groups, age and sex. For example, the First Nations population has the longest delay to primary care follow-up and the lowest specialist follow-up. Similar patterns have been seen in Alberta with respect to contact with pulmonary specialists following ED visits for asthma [21].

The estimated median time to the first follow-up visit was 5 days, shorter than for other chronic medical conditions such as asthma (19 days) [20] and COPD (13 days) [22]. Almost all patients are followed up at least once following their ED discharge; however, access to specialized cardiac services and consultation was variable. Moreover, many patients made multiple visits, likely reflecting a combination of the need for anticoagulation monitoring, repeat symptoms and/or complications. Finally, the outcomes for AFF presentations to the ED in Alberta are similar to those identified in a recent Ontario study [23].

There are limited similar studies available for comparison and their results differ from what we report. For example, the Ontario evaluation of atrial fibrillation cases [9] from 2002 to 2010 showed an increase in the crude rate of ED presentations for patients aged 18 to 105 years, whereas our crude rates remained relatively stable and even decreased slightly for patients aged ≥35. Alberta had lower admission rates than Ontario although this decreased in both provinces over time. The factors which might explain these observations are complex and may be related to that fact the databases employed do not contain more detailed information on other known risk factors for AFF (e.g., smoking status, diet), other health care resources outside the ED, and/or physician practices in ED and in primary care.

US data [8] from the National Hospital Ambulatory Medical Care Survey during 1993 to 2004 demonstrated an increase in the population-adjusted rate for ED presentations with a primary diagnosis of atrial fibrillation. Conversely, from 1999 to 2004, DSVRs in Alberta were generally stable with some slight increases. The admission proportion in patients with AFF in the US remained stable over time in this study and higher than similar patients in Canada, whereas our Alberta study saw decreases in admission. The reasons for these important differences require further exploration.

This study has several limitations. First, the databases used to describe AFF could not differentiate between recent-onset AFF and chronic AFF which caused symptoms severe enough to require an ED visit. Second, the databases accessed in this study are unable to capture all cases of AFF visits to the healthcare system, “true” incidence of disease, and the severity of the AFF episode. Third, patients were required to have the first recorded diagnosis be AFF and cases were randomly selected for study inclusion when multiple visits occurred in the year. Fourth, we fully recognize the definition of First Nations will underestimate the Aboriginal population. In Canada, there are three distinct Aboriginal peoples and these groups have been shown to be high users of the emergency health care system, especially for cardio-respiratory problems [21, 24]. In addition, the databases do not contain all potential confounding variables that might be able to explain observed differences in outcomes. Finally, it is not clear if all patients presenting to the ED with AFF need a cardiology follow-up visit and if the follow-up visits to cardiologists were directly linked with the preceding ED presentation.

## Conclusions

ED presentations of patients experiencing atrial fibrillation and flutter number over 5,000 annually in Alberta. While presentation rates across the province are stable, follow-up with physicians, consultation with cardiologists and health outcomes vary based on socio-economic, age, sex, and First Nations status. Further research is required to understand these inequalities.

## Additional file

Additional file 1: Three additional tables and two additional figures. Table S1. Sex and age group directly standardized visit rates per 1,000 population (aged ≥ 35) by fiscal year and subsidy group. Table S2. Frequency and percentage (%) of ED* visits for AFF† by triage level for each fiscal year. Table S3. Duration of ED* visits for AFF† by disposition status and major geographic areas for patients (≥35 years). Median (Med), 25th percentile (25th) and 75th percentile (75th) are provided. Figure S1. Sex and age group directly standardized visit rates per 1,000 population by fiscal year and subsidy group for those aged 35–64: First Nations (∆), Government Sponsored Programs (+), Human Services Recipient (×), and Other (○). Figure S2. Sex and age group directly standardized visit rates per 1,000 population by fiscal year and subsidy group for seniors: First Nations (∆) and non-First Nations (○). (DOCX 310 kb)

## Abbreviations

ACCS: Ambulatory Care Classification System
AFF: Atrial fibrillation and flutter
CI: Confidence interval
COPD: Chronic obstructive pulmonary disease
CTAS: Canadian Triage Acuity Scale
DSVR: Directly standardized visit rate
ED: Emergency department
ICD-10-CA: International Classification of Diseases, 10th Revision, Canadian Enhancement
ICD-9-CM: International Classification of Diseases, 9th Revision, Clinical Modification
IQR: Interquartile range
p: *P*-value
US: United States
