# Supplementary material for: Emergency department presentations for atrial fibrillation and flutter in Alberta: a large population-based study
Source: BMC Emerg Med. 2017 Jan 10;17:2. doi: 10.1186/s12873-016-0113-2 (PMC5223420; doi:10.1186/s12873-016-0113-2)
Supplement: Additional file 1: — Three additional tables and two additional figures. Table S1. Sex and age group directly standardized visit rates per 1,000 population (aged ≥ 35) by fiscal year and subsidy group. Table S2. Frequency and percentage (%) of ED* visits for AFF† by triage level for each fiscal year. Table S3. Duration of ED* visits for AFF† by disposition status and major geographic areas for patients (≥35 years). Median (Med), 25th percentile (25th) and 75th percentile (75th) are provided. Figure S1. Sex and age group directly standardized visit rates per 1,000 population by fiscal year and subsidy group for those aged 35–64: First Nations (∆), Government Sponsored Programs (+), Human Services Recipient (×), and Other (○). Figure S2. Sex and age group directly standardized visit rates per 1,000 population by fiscal year and subsidy group for seniors: First Nations (∆) and non-First Nations (○). (DOCX 310 kb) [file 12873_2016_113_MOESM1_ESM.docx]

**ADDITIONAL MATERIALS**

**Supplementary Table 1.** Sex and age group directly standardized visit rates per 1,000 population (aged ≥ 35) by fiscal year and subsidy group.

| Fiscal Year | First Nations aged 35-64‡ | Government Sponsored Programs aged 35-64 | Human Services Recipient 35-64 | Other aged 35-64 | First Nations  Seniors | Non-First Nations Seniors |
| --- | --- | --- | --- | --- | --- | --- |
| 1999/2000 | | | | | | |
| DSVR*(SD†) | 1.4 (0.3) | 1.4 (0.2) | 1.6 (0.3) | 1.1 (0.1) | 10.0 (2.3) | 9.9 (0.3) |
| 95% CI | (0.8, 2.0) | (0.9, 1.8) | (1.1, 2.1) | (1.0, 1.2) | (5.5, 14.6) | (9.4, 10.4) |
| 2000/2001 | | | | | | |
| DSVR(SD) | 1.7 (0.3) | 1.6 (0.2) | 2.3 (0.5) | 1.2 (0.1) | 12.1 (2.7) | 10.0 (0.3) |
| 95% CI | (1.0, 2.3) | (1.2, 2.0) | (1.4, 3.2) | (1.1, 1.3) | (6.8, 17.4) | (9.5, 10.5) |
| 2001/2002 | | | | | | |
| DSVR(SD) | 1.9 (0.3) | 1.6 (0.2) | 2.1 (0.4) | 1.2 (0.1) | 11.9 (2.8) | 10.2 (0.2) |
| 95% CI | (1.3, 2.6) | (1.2, 2.0) | (1.2, 3.0) | (1.1, 1.3) | (6.5, 17.4) | (9.7, 10.7) |
| 2002/2003 | | | | | | |
| DSVR(SD) | 2.0 (0.3) | 1.6 (0.2) | 1.8 (0.3) | 1.3 (0.1) | 16.1 (3.6) | 10.6 (0.3) |
| 95% CI | (1.3, 2.7) | (1.2, 1.9) | (1.2, 2.3) | (1.1, 1.4) | (8.9, 23.2) | (10.0, 11.1) |
| 2003/2004 | | | | | | |
| DSVR(SD) | 1.6 (0.3) | 1.3 (0.1) | 2.1 (0.3) | 1.2 (0.0) | 7.5 (2.1) | 10.4 (0.3) |
| 95% CI | (1.1, 2.1) | (1.0, 1.6) | (1.5, 2.7) | (1.1, 1.3) | (3.4, 11.6) | (9.9, 10.9) |
| 2004/2005 | | | | | | |
| DSVR(SD) | 1.6 (0.2) | 1.2 (0.1) | 1.3 (0.2) | 1.2 (0.1) | 8.6 (2.1) | 10.1 (0.2) |
| 95% CI | (1.1, 2.1) | (0.9, 1.5) | (0.9, 1.6) | (1.1, 1.3) | (4.5, 12.7) | (9.7, 10.6) |
| 2005/2006 | | | | | | |
| DSVR(SD) | 1.2 (0.2) | 1.7 (0.2) | 1.7 (0.2) | 1.3 (0.1) | 12.5 (3.0) | 10.5 (0.2) |
| 95% CI | (0.7, 1.7) | (1.3, 2.2) | (1.3, 2.2) | (1.2, 1.4) | (6.7, 18.3) | (10.0, 11.0) |
| 2006/2007 | | | | | | |
| DSVR(SD) | 1.7 (0.3) | 1.3 (0.1) | 1.3 (0.2) | 1.2 (0.0) | 12.6 (3.0) | 9.8 (0.2) |
| 95% CI | (1.2, 2.2) | (1.1, 1.6) | (0.9, 1.6) | (1.1, 1.3) | (6.8, 18.4) | (9.3, 10.2) |
| 2007/2008 | | | | | | |
| DSVR(SD) | 2.0 (0.4) | 1.2 (0.1) | 1.9 (0.3) | 1.1 (0.0) | 14.8 (3.2) | 9.4 (0.2) |
| 95% CI | (1.3, 2.8) | (1.0, 1.5) | (1.3, 2.5) | (1.0, 1.2) | (8.4, 21.1) | (8.9, 9.8) |
| 2008/2009 | | | | | | |
| DSVR(SD) | 1.8 (0.4) | 1.3 (0.2) | 1.5 (0.2) | 1.1 (0.0) | 10.9 (2.5) | 9.1 (0.2) |
| 95% CI | (1.0, 2.6) | (1.0, 1.7) | (1.1, 1.9) | (1.1, 1.2) | (6.1, 15.8) | (8.7, 9.5) |
| 2009/2010 | | | | | | |
| DSVR(SD) | 2.4 (0.4) | 2.0 (0.4) | 2.2 (0.3) | 1.1 (0.0) | 12.9 (2.6) | 8.9 (0.2) |
| 95% CI | (1.7, 3.1) | (1.2, 2.8) | (1.6, 2.7) | (1.0, 1.1) | ( 7.8, 17.9 ) | (8.5, 9.3) |
| 2010/2011 | | | | | | |
| DSVR(SD) | 2.3 (0.3) | 1.7 (0.3) | 1.5 (0.2) | 1.1 (0.0) | 11.5 (2.2) | 8.9 (0.2) |
| 95% CI | (1.6, 2.9) | (1.2, 2.2) | (1.1, 1.9) | (1.1, 1.2) | (7.2, 15.8) | (8.5, 9.3) |

*Directly Standardized Visit Rate

†Standard Deviation

‡Reference category is all Albertans aged 35 to 64 for columns based on ages 35 to 64, and all Albertans aged ≥65 for columns based on ages 65 and over.

**Supplementary Table 2.** Frequency and percentage (%) of ED* visits for AFF† by triage level for each fiscal year.

|  | N | Resuscitation  (CTAS‡  I) | | | Emergency  (CTAS II) | | Urgent  (CTAS III) | | Semi-urgent (CTAS IV) | | Non-urgent (CTAS V) | | Unavailable | | |
| --- | --- | --- | --- | --- | --- | --- | --- | --- | --- | --- | --- | --- | --- | --- | --- |
| Era of Optional Reporting | | | | | | | | | | | | | | |  |
| 1999/2000 | 4,262 | 19 | (0.4) | 192 | | (4.5) | 471 | (11.1) | 232 | (5.4) | 59 | (1.4) | 3,289 | (77.2) |  |
| 2000/2001 | 4,657 | 13 | (0.3) | 441 | | (9.5) | 811 | (17.4) | 174 | (3.7) | 92 | (2.0) | 3,126 | (67.1) |  |
| 2001/2002 | 4,834 | 15 | (0.3) | 550 | | (11.4) | 1,044 | (21.6) | 200 | (4.1) | 88 | (1.8) | 2,937 | (60.8) |  |
| 2002/2003 | 5,213 | 16 | (0.3) | 690 | | (13.2) | 1,381 | (26.5) | 325 | (6.2) | 308 | (5.9) | 2,493 | (47.8) |  |
| 2003/2004 | 5,222 | 31 | (0.6) | 1,195 | | (22.9) | 1,603 | (30.7) | 367 | (7.0) | 267 | (5.1) | 1,759 | (33.7) |  |
| Era of Mandatory Reporting for Urban Hospitals | | | | | | | | | | | | | | |  |
| 2004/2005 | 5,267 | 23 | (0.4) | 1,726 | | (32.8) | 1,924 | (36.5) | 426 | (8.1) | 328 | (6.2) | 840 | (15.9) |  |
| Era of Mandatory Reporting for Regional Hospitals | | | | | | | | | | | | | | |  |
| 2005/2006 | 5,735 | 21 | (0.4) | 2,127 | | (37.1) | 2,048 | (35.7) | 445 | (7.8) | 304 | (5.3) | 790 | (13.8) |  |
| Era of Mandatory Reporting for All Rural Hospitals | | | | | | | | | | | | | | |  |
| 2006/2007 | 5,567 | 26 | (0.5) | 2,159 | | (38.8) | 1,941 | (34.9) | 455 | (8.2) | 299 | (5.4) | 687 | (12.3) |  |
| 2007/2008 | 5,437 | 20 | (0.4) | 2,224 | | (40.9) | 1,936 | (35.6) | 517 | (9.5) | 274 | (5.0) | 466 | (8.6) |  |
| 2008/2009 | 5,568 | 48 | (0.9) | 2,298 | | (41.3) | 1,973 | (35.4) | 510 | (9.2) | 302 | (5.4) | 437 | (7.8) |  |
| 2009/2010 | 5,683 | 22 | (0.4) | 2,393 | | (42.1) | 2,126 | (37.4) | 564 | (9.9) | 277 | (4.9) | 301 | (5.3) |  |
| 2010/2011 | 5,953 | 21 | (0.4) | 2,516 | | (42.3) | 2,243 | (37.7) | 559 | (9.4) | 320 | (5.4) | 294 | (4.9) |  |

*Emergency Department

†Atrial Fibrillation/Flutter

‡Canadian Triage and Acuity Scale

**Supplementary Table 3.** Duration of ED* visits for AFF† by disposition status and major geographic areas for patients (≥35 years). Median (Med), 25^th^ percentile (25^th^) and 75^th^ percentile (75^th^) are provided.

|  | Edmonton Zone | | | Calgary Zone | | | All Other Zones | | | All Zones Combined | | |
| --- | --- | --- | --- | --- | --- | --- | --- | --- | --- | --- | --- | --- |
|  | Med | 25^th^ | 75^th^ | Med | 25^th^ | 75^th^ | Med | 25^th^ | 75^th^ | Med | 25^th^ | 75^th^ |
| Admitted | 7h 27m | 4h 4m | 12h 39m | 6h 51m | 4h 24m | 10h 19m | 2h 35m | 1h 26m | 4h 53m | 4h 16m | 2h 0m | 8h 14m |
| Discharged | 4h 56m | 3h 10m | 8h 0m | 4h 7m | 2h 41m | 6h 13m | 2h 13m | 1h 5m | 4h 14m | 3h 43m | 2h 2m | 6h 20m |
| All Dispositions Combined | 5h 14m | 3h 16m | 8h 39m | 4h 43m | 2h 56m | 7h 25m | 2h 21m | 1h 13m | 4h 30m | 3h 50m | 2h 1m | 6h 49m |

*Emergency Department

†Atrial Fibrillation/Flutter

**Supplementary Figure 1.** Sex and age group directly standardized visit rates per 1,000 population by fiscal year and subsidy group for those aged 35-64: First Nations (∆), Government Sponsored Programs (+), Human Services Recipient (×), and Other (○).

**
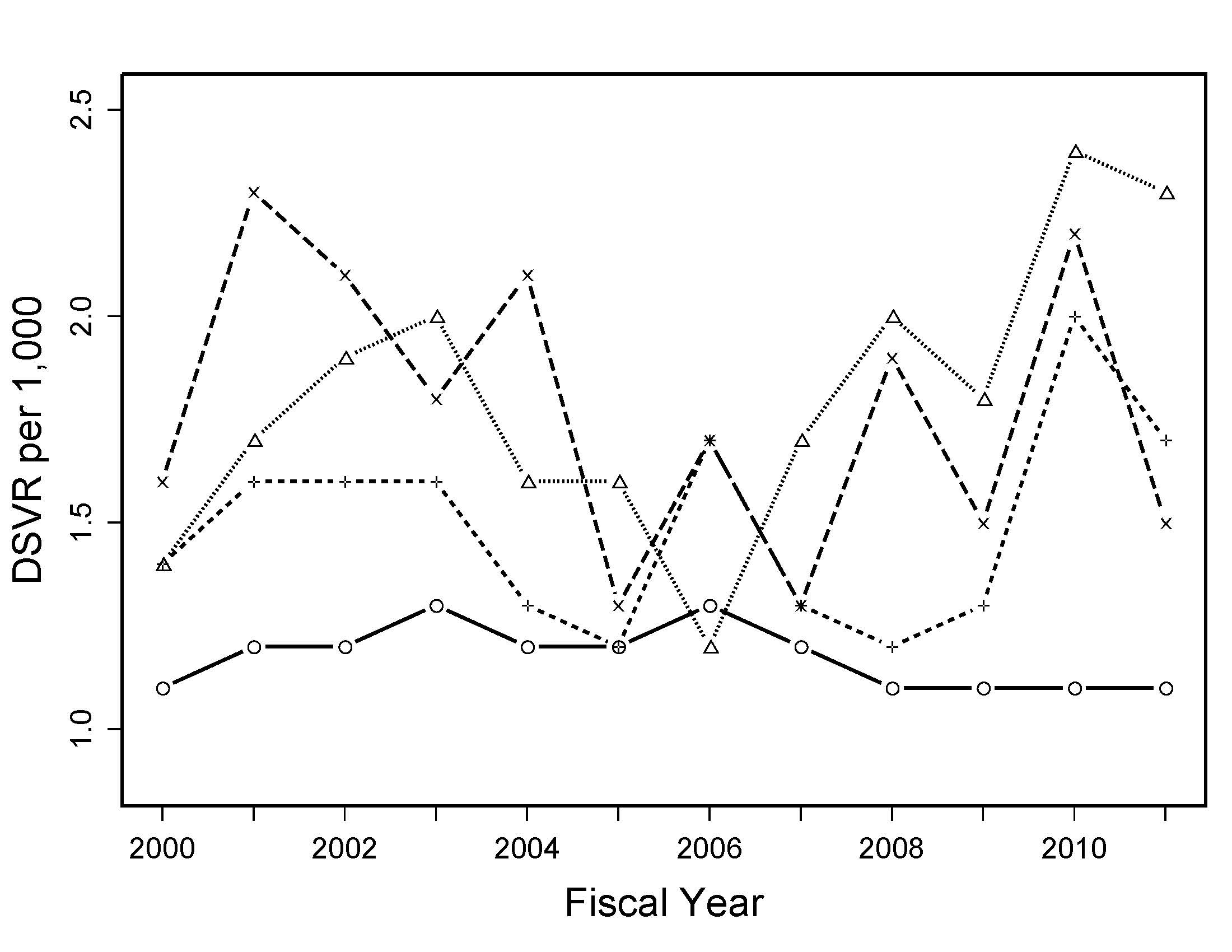
**

**Supplementary Figure 2.** Sex and age group directly standardized visit rates per 1,000 population by fiscal year and subsidy group for seniors: First Nations (∆) and non-First Nations (○).

**
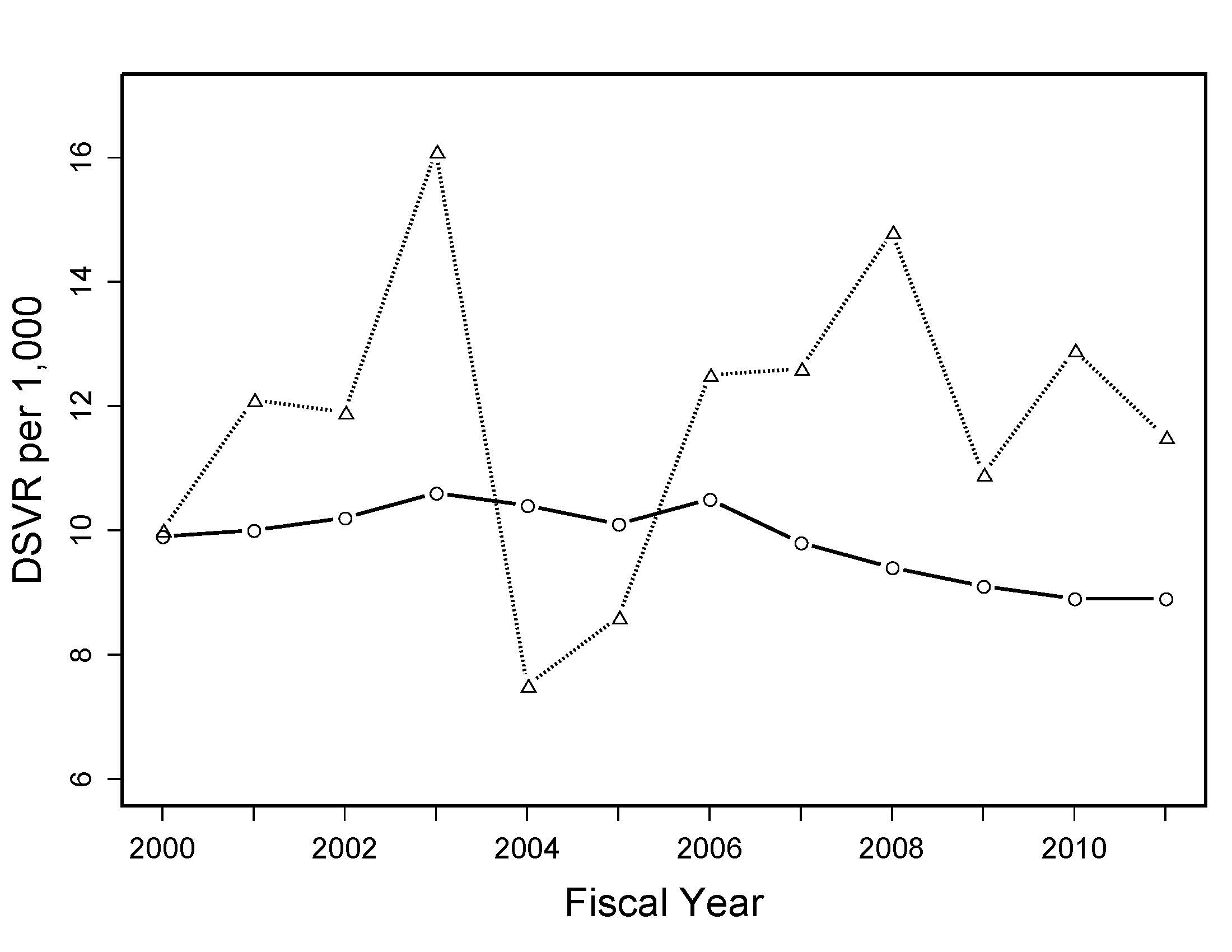
**
